# Supplementary figures and images for: Age, period, and cohort effects of Clonorchis sinensis infection prevalence in the Republic of Korea: Insights and projections
Source: PLoS Negl Trop Dis. 2024 Oct 11;18(10):e0012574. doi: 10.1371/journal.pntd.0012574 (PMC11498711; doi:10.1371/journal.pntd.0012574)

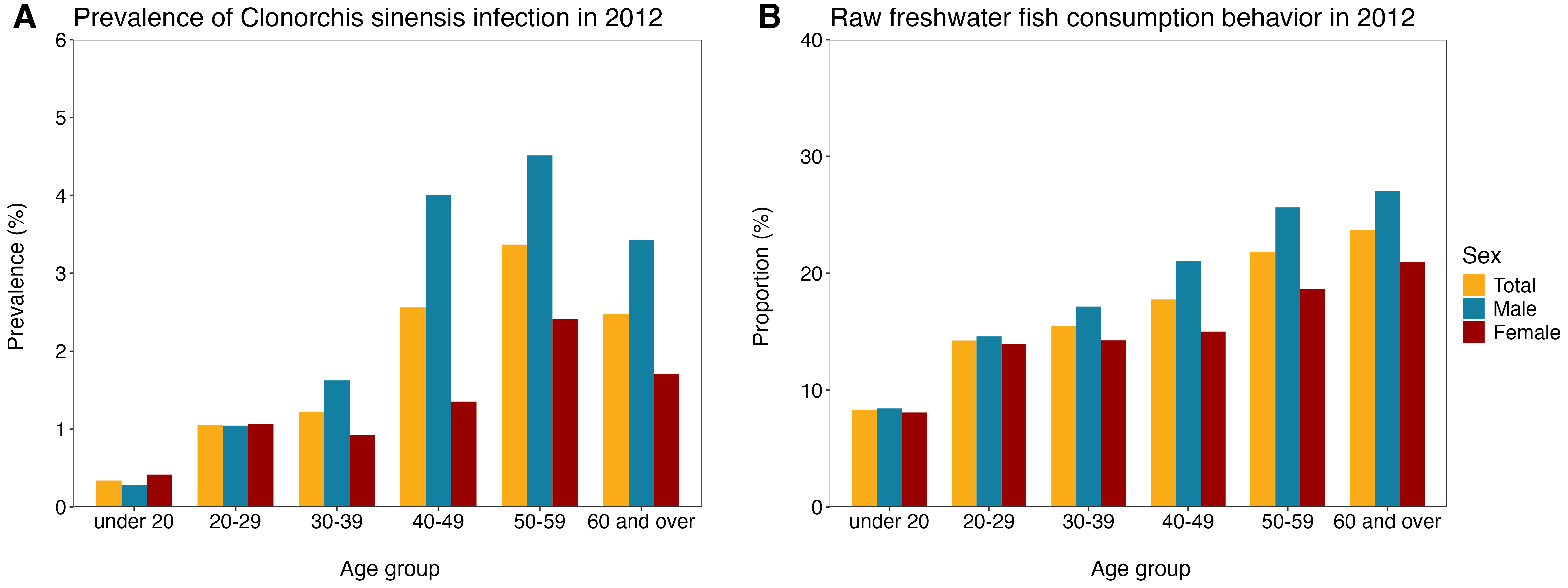

Supplement: S1 Fig — (TIF) [file pntd.0012574.s001.tif]

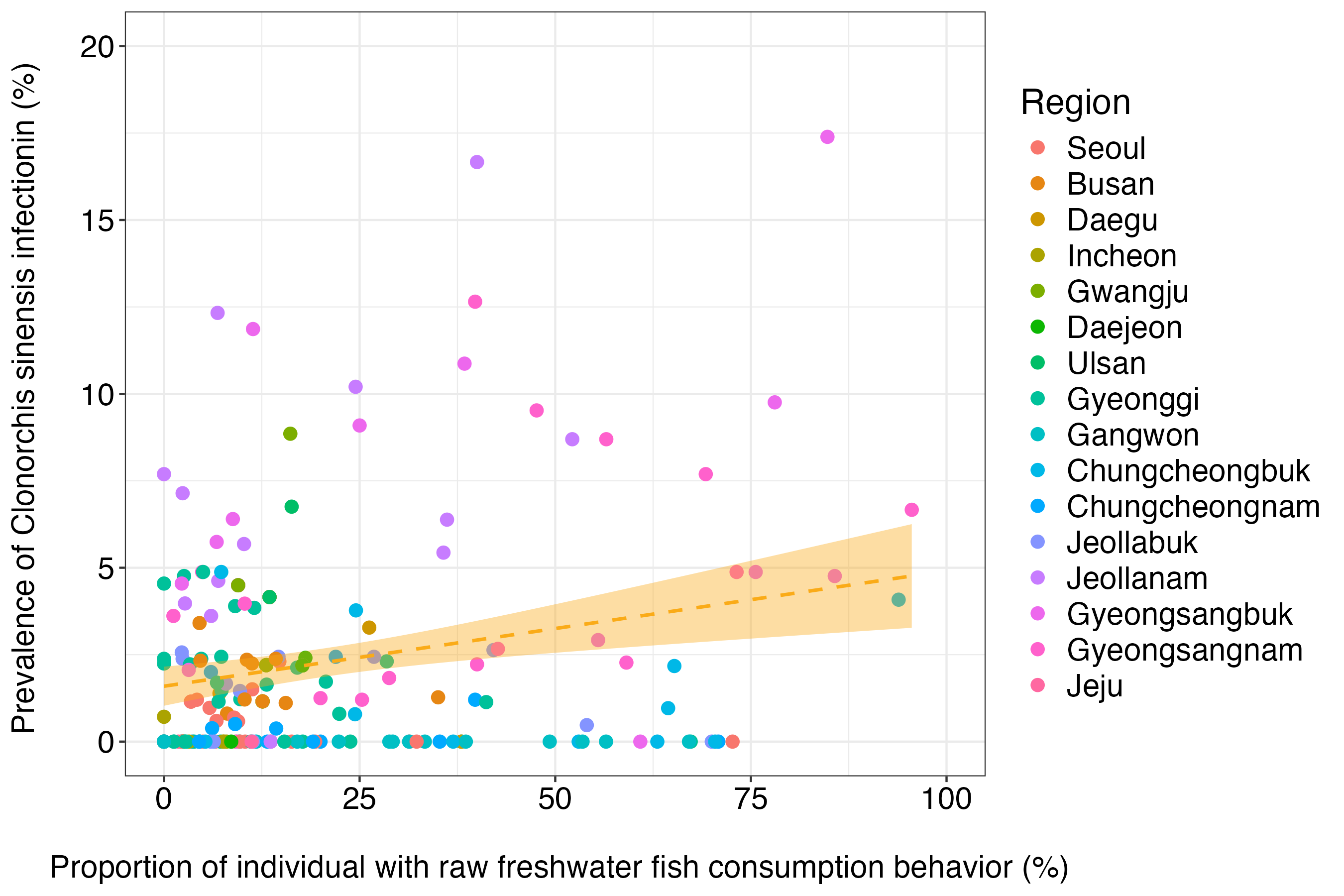

Supplement: S2 Fig — (TIF) [file pntd.0012574.s002.tif]
