# Supplementary material for: Age, period, and cohort effects of Clonorchis sinensis infection prevalence in the Republic of Korea: Insights and projections
Source: PLoS Negl Trop Dis. 2024 Oct 11;18(10):e0012574. doi: 10.1371/journal.pntd.0012574 (PMC11498711; doi:10.1371/journal.pntd.0012574)
Supplement: S1 Table — (DOCX) [file pntd.0012574.s003.docx]

**S1 Table. Details of national intestinal parasitic infections surveys from 1981–2012, the Republic of Korea**

| Survey year | 1981 | 1986 | 1992 | 1997 | 2004 | 2012 |
| --- | --- | --- | --- | --- | --- | --- |
| Survey regions | 104 census tracts based on 1980 census (ordinary, island) | Not reported | 194 ordinary census tracts from 1985 Population and Housing census | 203 ordinary census tracts from 1990 Population and Housing census | 300 census tracts from 2000 Population and Housing census | 602 census tracts from 2010 Population and Housing census |
| Sampling method | Not reported | Not reported | Stratified probability proportional sampling | Stratified robability proportional sampling | Stratified* probability proportional sampling | Stratified probability proportional sampling |
| Sample size  (Urban/rural proportion) | 40,119  (58% / 42%) | 47,671  (61% / 39%) | 51,556  (73% / 27%) | 49,977  (75% / 25%) | 22,828  (79% / 21%) | 24,423  (76% / 24%) |
| Egg counting techniques | Stoll’s dilution method | Stoll’s dilution method & Kato-Katz method | Kato-Katz method | Kato-Katz method | Kato-Katz method | Kato-Katz method |

*Individuals residing in endemic regions (areas with a high prevalence of *Clonorchis sinensis* infection) might be oversampled.
