# Supplementary material for: Age, period, and cohort effects of Clonorchis sinensis infection prevalence in the Republic of Korea: Insights and projections
Source: PLoS Negl Trop Dis. 2024 Oct 11;18(10):e0012574. doi: 10.1371/journal.pntd.0012574 (PMC11498711; doi:10.1371/journal.pntd.0012574)
Supplement: S2 Table — (DOCX) [file pntd.0012574.s004.docx]

**S2 Table. Details of intestinal parasitic infections surveys in residents by five major river streams from 2013–2021, the Republic of Korea***

| Survey year | 2013 | 2014 | 2015 | 2016 | 2017 | 2018 | 2019 | 2020 | 2021 |
| --- | --- | --- | --- | --- | --- | --- | --- | --- | --- |
| Number of survey regions** | 36 | 39 | 40 | 34 | 32 | 31 | 32 | 43 | 37 |
| Sample size | 38,739 | 41,139 | 42,024 | 34,943 | 38,648 | 32,792 | 30,415 | 25,642 | 21,410 |
| Cases positive for total parasites (%) | 3,694  (9.5) | 2,682  (6.5) | 2,195  (5.2) | 1,840  (5.3) | 1,927  (5.0) | 2,119  (6.5) | 1,528  (5.0) | 1,502  (5.9) | 1,116  (5.2) |
| Case positive for *Clonorchis sinensis* (%) | 2,950  (7.6) | 2,029  (4.9) | 1,778  (4.2) | 1,419  (4.1) | 1,524  (3.9) | 1,351  (4.1) | 944  (3.1) | 974  (3.8) | 699  (3.3) |

*The five major river streams include Han River, Geum River, Yeongsan River, Seomjin River, and Nakdong River.

**Specific survey regions within each river stream varied according to the voluntary participation of public health centers each year.
