# Supplementary material for: Age, period, and cohort effects of Clonorchis sinensis infection prevalence in the Republic of Korea: Insights and projections
Source: PLoS Negl Trop Dis. 2024 Oct 11;18(10):e0012574. doi: 10.1371/journal.pntd.0012574 (PMC11498711; doi:10.1371/journal.pntd.0012574)
Supplement: S3 Table — (DOCX) [file pntd.0012574.s005.docx]

**S3 Table. Model comparisons among all considered age-period-cohort models**

| Model | AIC* | Deviance | Degree of freedom |
| --- | --- | --- | --- |
| Age-period-cohort | 269.15 | 33.31 | 12 |
| Age-period | 325.11 | 105.27 | 20 |
| Age-cohort | 488.35 | 362.51 | 17 |
| Age-drift | 541.24 | 331.40 | 25 |
| Age | 744.00 | 536.16 | 26 |

*AIC: Akaike information criterion
