# Supplementary material for: Age, period, and cohort effects of Clonorchis sinensis infection prevalence in the Republic of Korea: Insights and projections
Source: PLoS Negl Trop Dis. 2024 Oct 11;18(10):e0012574. doi: 10.1371/journal.pntd.0012574 (PMC11498711; doi:10.1371/journal.pntd.0012574)
Supplement: S4 Table — (DOCX) [file pntd.0012574.s006.docx]

**S4 Table. Projected age-stratified prevalence of *Clonorchis sinensis* infection from 2013–2023, Republic of Korea**

| Age group | Year | Prevalence (90% projection intervals) |
| --- | --- | --- |
| 20–29 years | 2013 | 0.9% (0.7–1.0%) |
|  | 2014 | 0.8% (0.6–1.0%) |
|  | 2015 | 0.8% (0.6–1.0%) |
|  | 2016 | 0.7% (0.5–1.0%) |
|  | 2017 | 0.6% (0.4–1.1%) |
|  | 2018 | 0.6% (0.3–1.1%) |
|  | 2019 | 0.6% (0.3–1.1%) |
|  | 2020 | 0.5% (0.2–1.2%) |
|  | 2021 | 0.5% (0.2–1.2%) |
|  | 2022 | 0.5% (0.2–1.3%) |
|  | 2023 | 0.4% (0.1–1.4%) |
| 30–39 years | 2013 | 1.5% (1.3–1.7%) |
|  | 2014 | 1.4% (1.1–1.7%) |
|  | 2015 | 1.3% (1.0–1.7%) |
|  | 2016 | 1.2% (0.8–1.7%) |
|  | 2017 | 1.1% (0.7–1.8%) |
|  | 2018 | 1.0% (0.6–1.8%) |
|  | 2019 | 1.0% (0.5–1.9%) |
|  | 2020 | 0.9% (0.4–2.0%) |
|  | 2021 | 0.8% (0.3–2.1%) |
|  | 2022 | 0.8% (0.3–2.2%) |
|  | 2023 | 0.7% (0.2–2.3%) |
| 40–49 years | 2013 | 2.3% (2.0–2.7%) |
|  | 2014 | 2.2% (1.8–2.7%) |
|  | 2015 | 2.0% (1.6–2.7%) |
|  | 2016 | 1.9% (1.3–2.7%) |
|  | 2017 | 1.8% (1.1–2.7%) |
|  | 2018 | 1.6% (1.0–2.8%) |
|  | 2019 | 1.5% (0.8–2.9%) |
|  | 2020 | 1.4% (0.7–3.0%) |
|  | 2021 | 1.3% (0.5–3.2%) |
|  | 2022 | 1.2% (0.4–3.3%) |
|  | 2023 | 1.1% (0.4–3.5%) |
| 50–59 years | 2013 | 2.8% (2.4–3.2%) |
|  | 2014 | 2.6% (2.1–3.1%) |
|  | 2015 | 2.4% (1.8–3.1%) |
|  | 2016 | 2.2% (1.6–3.1%) |
|  | 2017 | 2.1% (1.3–3.2%) |
|  | 2018 | 1.9% (1.1–3.3%) |
|  | 2019 | 1.8% (1.0–3.4%) |
|  | 2020 | 1.7% (0.8–3.5%) |
|  | 2021 | 1.6% (0.7–3.7%) |
|  | 2022 | 1.4% (0.5–3.9%) |
|  | 2023 | 1.3% (0.4–4.1%) |
| 60 years and over | 2013 | 2.5% (2.2–2.9%) |
|  | 2014 | 2.3% (1.9–2.8%) |
|  | 2015 | 2.1% (1.6–2.8%) |
|  | 2016 | 2.0% (1.4–2.8%) |
|  | 2017 | 1.9% (1.2–2.8%) |
|  | 2018 | 1.7% (1.0–2.9%) |
|  | 2019 | 1.6% (0.9–3%) |
|  | 2020 | 1.5% (0.7–3.1%) |
|  | 2021 | 1.4% (0.6–3.2%) |
|  | 2022 | 1.3% (0.5–3.4%) |
|  | 2023 | 1.2% (0.4–3.6%) |
