# Supplementary material for: Age, period, and cohort effects of Clonorchis sinensis infection prevalence in the Republic of Korea: Insights and projections
Source: PLoS Negl Trop Dis. 2024 Oct 11;18(10):e0012574. doi: 10.1371/journal.pntd.0012574 (PMC11498711; doi:10.1371/journal.pntd.0012574)
Supplement: Attachment — Submitted filename: comments and suggestions - PLOS NTD.docx [file pntd.0012574.s007.docx]

**General comments and suggestions:**

*Clonorchis sinensis* (family Opisthorchiidae) is an important foodborne parasite that has a significant impact on the health of animals and humans. *C. sinensis* is mainly popular in China, South Korea, Vietnam, and the Russian Far East. This study applied the APC modeling approach to nationally representative time-series data from 1981-2012, and projected the age-stratified prevalence up to 2023. The findings are relatively explained. However, a revision is necessary and the specific comments are as follows.

**Specific comments:**

1. Authors has poor knowledge of the distribution of *Clonorchis sinensis.* Did *C. sinensis* only endemic to Korea, China and northern Vietnam? In addition, authors made serious mistake. Taiwan is one province of China, so the “Taiwan” should be deleted in this sentence in line 67. Please check and correct.
2. Why use the data from 1981-2012? And what’s the basis? After all, it has been too many years since then.
3. It’s 2024 now. While the study projected the age-stratified prevalence up to 2023 which is not real-time enough, and did not provide the latest data.
4. What’s the basis for dividing age groups? This should be described.
5. All tables should be shown as three-line.
