# Supplementary material for: Age, period, and cohort effects of Clonorchis sinensis infection prevalence in the Republic of Korea: Insights and projections
Source: PLoS Negl Trop Dis. 2024 Oct 11;18(10):e0012574. doi: 10.1371/journal.pntd.0012574 (PMC11498711; doi:10.1371/journal.pntd.0012574)
Supplement: Attachment — Submitted filename: Responses to the reviewers.docx [file pntd.0012574.s008.docx]

***Responses to Reviewer #1***

Clonorchis sinensis (family Opisthorchiidae) is an important foodborne parasite that has a significant impact on the health of animals and humans. C. sinensis is mainly popular in China, South Korea, Vietnam, and the Russian Far East. This study applied the APC modeling approach to nationally representative time-series data from 1981-2012, and projected the age-stratified prevalence up to 2023. The findings are relatively explained. However, a revision is necessary and the specific comments are as follows.

>> We thank the reviewer for the time and effort in providing valuable comments.

1. Authors has poor knowledge of the distribution of Clonorchis sinensis. Did C. sinensis only endemic to Korea, China and northern Vietnam? In addition, authors made serious mistake. Taiwan is one province of China, so the “Taiwan” should be deleted in this sentence in line 67. Please check and correct.

>> We thank the reviewer for the correction. We have now replaced the term “*country*” with “*region*” and “*China*” with “*mainland China*” in the revised manuscript following this comment. However, according to published literature, while other species of liver fluke (e.g., F. hepatica or O. felineus) are known to be endemic in various regions, *C. sinensis* is primarily endemic to Korea, southern and northeastern China, and northern provinces of Vietnam [1–4]. Additionally, we would like to clarify that the phrase “*primarily endemic to the East Asia region*” is meant to indicate that this region is a major area of endemicity, rather than implying that it is the exclusive region affected. Hence, we have opted to maintain the original phrase, while we have further clarified that those regions are not the only ones experiencing *C. sinensis* by adding “including” to the revised manuscript.

*[P4 L66–67] “Clonorchis sinensis (C. sinensis), a prevalent intestinal helminth primarily endemic to the East Asian region—including the Republic of Korea (hereafter “Korea”), mainland China, Taiwan, and northern Vietnam—poses significant public health threats in this region.”*

[1] Qian, Men-Bao, et al. "Clonorchiasis." The Lancet 387.10020 (2016): 800-810.

[2] Zheng, Jin-Xin, et al. "Natural variables separate the endemic areas of Clonorchis sinensis and Opisthorchis viverrini along a continuous, straight zone in Southeast Asia." Infectious diseases of poverty 13.02 (2024): 39-51.

[3] Hong, Sung-Tae, and Yueyi Fang. "Clonorchis sinensis and clonorchiasis, an update." Parasitology International 61.1 (2012): 17-24.

[4] US CDC Yellow Book 2024: https://wwwnc.cdc.gov/travel/yellowbook/2024/infections-diseases/flukes-liver#epi

2. Why use the data from 1981-2012? And what’s the basis? After all, it has been too many years since then.

>> We appreciate the reviewer for raising this point. We have not been clear enough about the available data points on the national survey of intestinal parasitic infection prevalence in Korea. To provide more detail, these surveys were conducted periodically from 1981 to 2012, with intervals of 5–8 years, however, no additional nationwide surveys have been conducted since 2012. Instead, the Korean government initiated an annual surveillance in 2011 focusing solely on residents in high-endemic areas.

We believe that relying exclusively on data from the high-endemic region survey, which covers a more recent but very limited timeframe and geographical area, poses challenges in assessing the cohort effect (which is the main focus of our study), as disentangling period and cohort effects often require a sufficient length of data [1]. Furthermore, combining these two datasets is not appropriate due to the different sampling designs and characteristics of the participant groups (i.e., nationally representative vs. high-endemic region residents). Hence, we opted to use the national survey data from 1981 to 2012 as the main dataset for our analyses, while validating the overall trend of projections using the high-endemic region survey data. We have now clearly clarified this point in the revised manuscript.

*[P7 L137–140] “Age-stratified prevalence of C. sinensis infection in Korea from 1981–2012 (specifically 1981, 1986, 1992, 1997, 2004, and 2012) was extracted from national surveys on the prevalence of intestinal parasitic infections conducted by the Korea Disease Control and Prevention Agency (KDCA), with the last survey conducted in 2012.”*

*[P9 L185–196] “To assess the plausibility of our projections, we compared them with the observed C. sinensis infection prevalence from 2013–2021 (covering the period from the start of the projections to the most recent data available) among residents in high-endemic areas of intestinal parasitic infections across Korea. This time-series data, sourced from a published paper (for 2013– 2020) and supplemented with non-public data from the KDCA (for 2021), comprises positive rates of the disease obtained during annual active screening campaigns conducted in 30–40 cities, primarily located near the 5 main river streams in Korea. While this data covers a more recent period than the data used in the analysis (covering 1981–2012), it was used exclusively for validating the overall trend of projections due to (i) its limited timeframe and geographical area, which restrict accurate assessments of the cohort effects, and (ii) the inability to combine it with our main data, given the differing sampling designs and participant group characteristics (i.e., nationally representative versus high-endemic region residents).”*

[1] Dinas, Elias, and Laura Stoker. "Age-Period-Cohort analysis: A design-based approach." Electoral Studies 33 (2014): 28-40.

3. It’s 2024 now. While the study projected the age-stratified prevalence up to 2023 which is not real-time enough, and did not provide the latest data.

>> As noted in comment #2, since the last available data point from the national survey is from 2012, we projected age-specific prevalence up to 2023, covering the next 10 years beyond the most recent data. We believe extending the projection further than 10 years could introduce excessive uncertainty and result in very wide projection intervals, which is less informative in public health planning. Despite our projection does not extend into future periods due to the constrained timeframe of our data, we have chosen to retain the term "*projection*" in our manuscript, because the APC modeling framework remains capable of projecting future disease trajectories, if recent data is available [1–2]. We have clarified this point in the revised manuscript.

*[P9 L180–L183] “Annual age-stratified prevalences were projected up to 2023, covering the next 10 years beyond the last available data point (2012), based on the national prospective population projections [23] and the posterior distribution of each effect obtained by fitting the model to the retrospective time-series data.”*

*[P15 L326–L329] “In addition, while our projections do not extend into the future (2013–2023) due to the data constraints, it should be noted that the APC modeling approach remains applicable for projecting future trajectories of the disease once updated data becomes available.”*

[1] Simões, Taynãna César, et al. "Chagas disease mortality in Brazil: A Bayesian analysis of age-period-cohort effects and forecasts for two decades." PLoS neglected tropical diseases 12.9 (2018): e0006798.

[2] Zhou, Jia-Li, et al. "Trends and projections of inflammatory bowel disease at the global, regional and national levels, 1990–2050: a bayesian age-period-cohort modeling study." BMC Public Health 23.1 (2023): 2507.

4. What’s the basis for dividing age groups? This should be described.

>> The age groups were categorized into 10-year bands, while (i) excluding those under 19 considering their negligible prevalence of *C. sinensis* infection throughout the study period, and (ii) those aged 60 and over were combined into a single group given their relatively small population size in the 10-year age bands for individuals over 60. We have now provided a detailed description of the age group categorization in the revised manuscript.

*[P7 L150–153] “All data were categorized into five age groups with 10-year bands: 20–29, 30–39, 40–49, 50–59, and 60 years and over (60+). Individuals under 19 were excluded due to their negligible prevalence of C. sinensis throughout the study period, and those aged 60+ were combined into a single group given their relatively small sample size.”*

5. All tables should be shown as three-line.

>> In response to this comment, all supplementary tables are now formatted with the three-line structure.

***Responses to Reviewer #2***

The manuscript use published data and generate new analysis in particular with the cohort factor. It is a brief research note. Here below are some key points needed to be addressed in the revised version.

>> We thank the reviewer for providing valuable comments to improve our manuscript.

1. The manuscript did not mention the actual size of investigated population that included in the analysis, which should be provided in the revised version.

>> The sample size for each nationwide survey (the main data for our analyses) has been listed in **Table S1**. Additionally, a new supplementary table (**Table S2**) has been added that summarizes the characteristics (including sample size) of the high-endemic region surveys, which were used to validate the overall trend of projections presented in **Fig. 3**.

2. Fig 3, there is one blue line, but two shadows (light blue and gray) in each panel. It seems that gray shadows are not described, suggested to remove them. Then, you could zoom in on the Y axis.

>> We apologize for the confusion. The blue shades in **Fig. 3** represent the 50% projection interval, while the lighter blue shades (referred to as the gray shade in this comment) represent the 90% projection interval. We have now corrected the figure legend to include all corresponding information.

*[Figure legend of* ***Fig.3****] “Blue lines indicate the median of the projected outcomes, while blue and lighter blue shades represent their 50% and 90% projection intervals, respectively.”*

3. Line 67, "countries", "China", "Taiwan". When mentioning "countries", it is inproper to put China and Taiwan on the same position, you may use"regions, including... Chinese mainland, Taiwan...". or "countries, including... China (with Taiwan province)...". as well as at Line298 (e.g., China, Taiwan...)

>> We thank the reviewer for pointing it out. We have now replaced the term “*country*” with “*region*” and “*China*” with “*mainland China*” in the revised manuscript following this comment.

*[P4 L66–67] “Clonorchis sinensis (C. sinensis), a prevalent intestinal helminth primarily endemic to the East Asian region—including the Republic of Korea (hereafter “Korea”), mainland China, Taiwan, and northern Vietnam—poses significant public health threats in this region.”*

*[P14 L314–P15 L317] “Nevertheless, this APC modeling approach holds broad applicability to other endemic regions to C. sinensis infection (e.g., mainland China, Taiwan, and northern Vietnam), serving as an effective tool for evaluating interventions and facilitating projections (or nowcasting) between survey iterations.”*

4. Line 93, "decline in national prevalence to 1.9% by 2012", Is this 1.9% only for C. sinensis? Maybe the following way is more clear, "...prevalence to 1.9% by 2012 for C. sinensis, comprising 73% of the total positivity rate for all intestinal parasitic infections, the country continues to face areas with high endemicity".

>> We thank the reviewer for the suggestion. To ensure clarity, this sentence intended to indicate that Korea continues to have high-endemic regions of *C. sinensis* infection, where the disease comprises more than 70% of the total positivity rate for all reported intestinal parasitic infections [1]. We have now clarified that the national prevalence specifically refers to *C. sinensis* infection in response to the reviewer’s comment.

*[P6 L95–96] “Despite these sustained efforts and a further decline in national prevalence to 1.9% by 2012, the country continues to face areas with high endemicity, where C. sinensis infection comprises more than 70% of the total positivity rate for all reported intestinal parasitic infections.”*

[1] Korea Centers for Disease Control and Prevention. Korea Association of Health Promotion: Prevalence of intestinal parasites in Korea - The 8th Report. Seoul, Korea: 2012.

5. In the case of older persons have higher infection proportion, did authors exclude the possibility effect of accumulation of raw fish consumption?

>> We appreciate the reviewer for raising this point. We agree that the higher prevalence among older populations could be partially attributed to previous exposure to *C. sinensis* following the accumulation of raw fish consumption. However, to clarify, given the widespread screenings and follow-up antiparasitic treatments implemented in Korea throughout the study period [1–2], we assume that the reported prevalence primarily reflects newly infected cases (incidence) in our study. Additionally, such effect of accumulated raw fish consumption is implicitly accounted for in our projections via the gradually increasing age effect (**Fig. 2A**), which likely reflects both the accumulation of exposure and the increasing likelihood of new exposure with aging. We have now addressed these points in the Limitations section of the revised manuscript.

*[P16 L354–P17 361] “First, our analysis relies on the assumption that the reported prevalence rate of C. sinensis infection is equivalent to the incidence rate, under the premise of widespread screening campaigns and subsequent follow-up treatment in Korea throughout the study period. However, the robustness of this assumption may be compromised if factors such as incomplete treatment or the accumulation of exposure to C. sinensis significantly affect the observed prevalence. Nonetheless, we believe that such effects are implicitly considered in our projections via the age effect (****Fig. 2A****), which likely reflects both the accumulation of exposure and the increasing likelihood of new exposure with aging.”*

[1] Yoo, Won Gi, Woon-Mok Sohn, and Byoung-Kuk Na. "Current status of Clonorchis sinensis and clonorchiasis in Korea: epidemiological perspectives integrating the data from human and intermediate hosts." Parasitology 149.10 (2022): 1296-1305.

[2] Kim, Hwa Sun, et al. "Relationship between Clonorchis sinensis infection and cholangiocarcinoma in Korea." The Korean journal of parasitology 60.4 (2022): 261.

6. Discussion should highlight the important effect of widespread allocation of praziquantel since 1983 and the government elimination program since 2005, which reform the trend of going down.

>> We thank the reviewer for the suggestion to improve our manuscript. We agree that we have not clearly addressed the potential effects of the nationwide application of praziquantel since 1983, while the reinstatement of the national elimination program from 2005 has been discussed in the manuscript. We have now added sentences to the Results and Discussion sections to further emphasize the role of interventions in Korea.

*[P11 L229–235] “The estimated prevalence ratio driven by period effect generally declined over the survey years (Fig 2C). This decreasing trend, after adjusting for the contribution of the cohort effect, suggests the effectiveness of interventions in Korea, including sustained non-pharmaceutical measures and the widespread application of praziquantel since 1983. Notably, fluctuations observed in 1997 and 2004, coincided with the suspension and subsequent reinstation of the nationwide eradication campaign in Korea in 1995 and 2005, respectively.”*

*[P13 L279–283] “In addition to the contribution of the cohort effect, our APC analysis showed a significant reduction in the risk of C. sinensis infection associated with the period effect. This suggests the potential effectiveness of both national-level interventions (1969–1995), including the nationwide application of praziquantel starting in 1983, and targeted interventions implemented in endemic regions (since 2005) in controlling C. sinensis infection in Korea.”*

7. Author may discuss: the younger generation (those born in the cohort of 1952 or later) that consuming less raw fish may largely influence by food imported from overseas, and having a keen interest in exploring new eating cultures.

>> We thank the reviewer for this comment. We agree that the increasing intake of a Westernized diet among younger generations [1] has also contributed to the lower consumption of raw freshwater fish in this group, along with school-based health promotion (aiming at changing risk behaviors of intestinal helminth infection) and familial dietary assimilation [2]. However, due to the scarcity of direct evidence linking these two factors, such as specific results from questionnaires, we have discussed its possibility in the revised manuscript as follows:

*[P12 L266–P13 270] “Consequently, the younger generation (those born in the cohort of 1952 or later) is likely to show lower engagement in consuming raw freshwater fish, whereas the older generation might sustain their dietary practices. Such generational differences in raw freshwater fish consumption might be further accentuated by the increasing adoption of Westernized diets among younger generations, while also being influenced by familial dietary assimilation.”*

[1] Park, Sohyun, et al. "Environmental influences on youth eating habits: insights from parents and teachers in South Korea." Ecology of food and nutrition 53.4 (2014): 347-362.

[2] Qian, Men-Bao, et al. "Familial assimilation in transmission of raw-freshwater fish-eating practice leading to clonorchiasis." PLoS neglected tropical diseases 14.4 (2020): e0008263.

***Responses to Reviewer #3***

This research applied the age-period-cohort modeling approach to nationally representative C. sinensis infection prevalence data from 1981–2012 in Korea, and the model was used to predict the incidence in the next few years. Overall, this study has certain novelty and practical value. However, some questions need to be addressed or answered.

>> We appreciate the reviewer for supporting the novelty and practical implication potential of our study.

1. The 'Age-period-cohort modeling' should be emphasized, including which references were made, how specific calculations were made, and what each coefficient represents.

>> We thank the reviewer for this comment. The mentioned aspects have been covered in the Introduction and Methods sections, and we have clarified these points more explicitly in the revised manuscript.

*[P6 L120–124] “In this framework, age effects reflect the physiological changes individuals undergo across their lifespan, while period effects signify simultaneous shifts affecting all age groups, often precipitated by specific events such as interventions. The cohort effects capture the collective influence of age and period effects, revealing variations among individuals born in the same year.”*

*[P8 L163–169] “We then modeled prevalence λ at age group a in survey calendar year p for those in birth cohort c as follows: log(λ(a,p))=f(a)+g(p)+h(c), where cohort c is defined as the difference between the start of the age group intervals and the calendar year of surveys (c=p-a). To address a non-identifiability issue arising from the linear dependency between these effects, the age-period (or age-cohort) model was first fitted, and then the logarithm of the fitted values was employed as an offset variable in a cohort effect (or period effect) model.”*

2. Lines 230-233 The data sources for 2015-2021 should be supported by literature, or relevant tables should be listed in the attachment. Why is there no relevant data for 2013 and 2014?

>> We apologize for the confusion. In response to this comment, we have cited a published paper that reports the age-specific prevalence of *C. sinensis* infection in endemic regions of Korea, based on the same data we used [1]. However, since this paper covers data only up to 2020, we incorporated non-public data provided by KDCA for 2021. In accordance with KDCA data policy, all requests for access to this non-public data should be directed to the corresponding authors and will be reviewed by the KDCA upon reasonable request. The corresponding description of the data has been corrected in the revised manuscript. Additionally, we have updated **Fig. 3** (and its legend) to include data from 2013 to 2021 (used for validating projections based on the national survey data available up to 2012), covering the start of the projection period (2013) up to the most recent data available (2021).

*[P9 L185–191] “To assess the plausibility of our projections, we compared them with the observed C. sinensis infection prevalence from 2013–2021 (covering the period from the start of the projections to the most recent data available) among residents in high-endemic areas of intestinal parasitic infections across Korea. This time-series data, sourced from a published paper (for 2013–2020) [29] and supplemented with non-public data from the KDCA (for 2021), comprises positive rates of the disease obtained during annual active screening campaigns conducted in 30–40 cities, primarily located near the 5 main river streams in Korea* ***(Table S2****).”*

[1] Lee, Myoung-Ro, et al. "Status of helminthic infections in residents around river basins in the Republic of Korea for 10 Years (2011–2020)." The Korean journal of parasitology 60.3 (2022): 187.

3. Is there a comparison and discussion between this model and similar models that have already been publicly published?

>> We thank the reviewer for raising this point. To be more precise, our age-period-cohort modeling analysis suggests that in the context of *C. sinensis* infection, where infection risk varies by birth cohort due to generational differences in raw freshwater fish consumption, the cohort effect significantly influences the time-varying transmission trends of the disease. This indicates that neglecting such cohort effects could result in biased (overestimated) estimates of intervention effects. Notably, to our knowledge, other published studies, particularly those focusing on the Korean context [1–3], have relied on models not explicitly incorporating such cohort effects.

In light of this, we believed that a simple comparison with studies without considering the cohort effect may lead to a misinterpretation of our main message. This is because while the overall conclusion remains consistent (i.e., significant impacts of interventions), the magnitude of the impact could be overestimated when the potential impact of the cohort effect is neglected. Furthermore, comparing the magnitude of the impact across different studies is often challenging due to variations in statistical approaches and resulting outcomes. Hence, rather than comparing our results with other published studies, we have addressed this point in the Discussion section of the revised manuscript (please also see our response to Reviewer 4’s comment #3).

*[P15 L331–P16 339] “Caution should be advised when comparing our results with studies not incorporating cohort effects. Although the overall significance of intervention effects might appear consistent, their magnitude could be overestimated if the cohort effects are not explicitly considered. This is particularly relevant in the context of C. sinensis infection, where infection risk varies significantly by birth cohort due to generational differences in raw freshwater fish consumption. Furthermore, variations in epidemiological and ecological conditions across countries (e.g., differences in intervention target groups, generational freshwater fish consumption patterns, and the dynamics of intermediate hosts), combined with differing statistical approaches, can further complicate direct comparisons between studies.*”

[1] Xiao, Hai-Yan, et al. "The spatial-temporal risk profiling of Clonorchis sinensis infection over 50 years implies the effectiveness of control programs in South Korea: a geostatistical modeling study." The Lancet Regional Health–Western Pacific 33 (2023).

[2] Oh, Jin‐Kyoung, et al. "Control of clonorchiasis in K orea: effectiveness of health education for community leaders and individuals in an endemic area." Tropical Medicine & International Health 19.9 (2014): 1096-1104.

[3] Kim, Chunmi, et al. "A study on the prevalence of clonorchis sinensis and the effects of educational program among residents in the basin of the Youngsan River, Korea." Journal of Korean Academy of Community Health Nursing 22.1 (2011): 56-65.

4. Line 67, Line 298: Please refer to the literature to correctly explain the relations between China and Taiwan（For example, doi: 10.1016/S0140-6736(15)60313-0.	doi: 10.1016/j.biotechadv.2018.02.008.）

>> We thank the reviewer for the suggestion to improve our manuscript. Both papers are now cited in the revised manuscript in their respective contexts.

5. Fig 3: What the light and dark shades of blue represent？

>> We apologize for the confusion. The blue shades in **Fig. 3** represent the 50% projection interval, while the lighter blue shades represent the 90% projection interval. We have now corrected the figure legend to include all corresponding information.

*[Figure legend of* ***Fig.3****] “Blue lines indicate the median of the projected outcomes, while blue and lighter blue shades represent their 50% and 90% projection intervals, respectively.”*

6. Lines 230-233: It was observed that the incidence of Fig3D and E was increased in the high age group, which is not consistent with the result description.

>> To ensure clarity, these panels show that the overall trend of age-specific projections (represented by the median value of the projections; solid blue lines) decreases over time, Meanwhile, the projection intervals (blue shades) become widen due to increased uncertainties as the projection period extends, rather than not indicating an increasing trend of projected prevalences.

***Responses to Reviewer #4***

1. Methods applied to existing data seem appropriate. As no new data was collected, the ethical considerations of collecting survey data were not necessary to mention.

>> We thank the reviewer for this comment. To clarify, the national survey data from 2012 (used to extract information on age-specific raw freshwater fish consumption behavior for **Fig. S1**) and the high-endemic region survey data from 2013 to 2021 (used to validate the direction of our projections in **Fig. 3**) were obtained from individual-level data. In addition, although we have now cited a published study reporting age-specific prevalences from the same high-endemic region data, this study does not include data from 2021. To address this gap and comply with the recommendations of our institutions' ethics committees for using individual-level data, we obtained an ethical approval waiver (as both datasets were anonymized at every stage), and this is currently noted in the Ethics statement section.

2. Results are described clearly and appropriately.

>> We appreciate the reviewer for supporting the appropriateness of our Results section.

3. Conclusions support intervention strategies used in the past and in the present. Some additional comparison with similar data from China for clonorchiasis would be useful to the reader and further support their conclusions.

>> We thank the reviewer for highlighting this issue. Our study emphasizes the importance of incorporating cohort effects when evaluating intervention strategies for controlling *C. sinensis* infection, as infection risk varies by birth cohort due to generational differences in raw freshwater fish consumption. However, to our knowledge, we observed that studies focusing on the Korean context have not explicitly incorporated the cohort effects in their models [1–3], thus, we believe that a simple comparison (whether the intervention effect was significant or not) could lead to a misinterpretation of our main message (please see our response to the Reviewer 3’s comment #3 for further discussions). Moreover, considering different epidemiological and ecological conditions between countries—including variations in factors (i) affecting dynamics in humans (e.g., intervention target groups [4], generational fish consumption behaviors) and (ii) nature dynamics of intermediate hosts (e.g., their inhabitant areas [5])—directly comparing trends across countries presents additional challenges. Hence, rather than comparing our results with other published studies, we have now addressed this clarification in the revised manuscript.

*[P15 L331–P16 339] “Caution should be advised when comparing our results with studies not incorporating cohort effects. Although the overall significance of intervention effects might appear consistent, their magnitude could be overestimated if the cohort effects are not explicitly considered. This is particularly relevant in the context of C. sinensis infection, where infection risk varies significantly by birth cohort due to generational differences in raw freshwater fish consumption. Furthermore, variations in epidemiological and ecological conditions across countries (e.g., differences in intervention target groups, generational freshwater fish consumption patterns, and the dynamics of intermediate hosts), combined with differing statistical approaches, can further complicate direct comparisons between studies.”*

[1] Xiao, Hai-Yan, et al. "The spatial-temporal risk profiling of Clonorchis sinensis infection over 50 years implies the effectiveness of control programs in South Korea: a geostatistical modeling study." The Lancet Regional Health–Western Pacific 33 (2023).

[2] Oh, Jin‐Kyoung, et al. "Control of clonorchiasis in Korea: effectiveness of health education for community leaders and individuals in an endemic area." Tropical Medicine & International Health 19.9 (2014): 1096-1104.

[3] Kim, Chunmi, et al. "A study on the prevalence of clonorchis sinensis and the effects of educational program among residents in the basin of the Youngsan River, Korea." Journal of Korean Academy of Community Health Nursing 22.1 (2011): 56-65.

[4] Qian, Men-Bao, and Xiao-Nong Zhou. "Children education for sustainable control of liver fluke infections." Infectious Diseases of Poverty 11.06 (2022): 90-93.

[5] Liu, Kai, et al. "Spatio-temporal disparities of Clonorchis sinensis infection in animal hosts in China: a systematic review and meta-analysis." Infectious Diseases of Poverty 12.05 (2023): 1-31.

4. The study depends heavily on available datasets that are more than ten years old and they use more recent survey data from high prevalence regions to assess the results of their models. Similar studies have been conducted in China for C. sinensis but similarities in trends observed were not compared/contrasted herein. In particular, how more recent geospatial data in China compares to the correlations observed in prevalance vs. region herein. Some bias in sampling regions with recent comparative data was inevitable due to the reduced prevalance, but should be discussed, perhaps? The results of the article support the importance of considering age group/cohorts when designing intervention strategies. The authors have not provided any changes/modifications from existing intervention methods (education programmes, targeted treatment to reduce parasite load etc.); only that is appears to work well

>> We thank the reviewer for the suggestions to improve our manuscript. In response to comments #3 and #4, we have now discussed the potential challenges of directly comparing our results with data from other countries in the revised manuscript (please refer to the detailed discussion in our response to comment #3).

We also agree that the implementation of targeted interventions with the consideration of age (or cohort) aspects is an important discussion point, which might lead to an underestimation of intervention effects in our APC modeling approach. In response to this comment, we have now discussed these points in the revised manuscript (please also see our response to comment #7).

5. Author summary is very similar to the abstract. It would be good to review to address accessibillity for a broader audience.

>> We thank the reviewer for the suggestion to improve our manuscript. We have now revised the Author Summary to distinguish it from the Abstract while maintaining the key messages of the study.

*[Author Summary] “Clonorchis sinensis (C. sinensis) infection, a neglected chronic parasitic infection, continues to be a significant public health concern with severe implications. Despite ongoing containment efforts leading to a decline in prevalence in Korea, assessing interventions remains challenging, due to variations in infection risk across different birth cohorts, driven by generational differences in raw freshwater fish consumption. We applied the age-period-cohort modeling approach to nationally representative C. sinensis infection prevalence data from 1981–2012 in Korea. Our analysis showed both cohort and period effects have significantly contributed to the observed decline in the prevalence of C. sinensis infection in Korea throughout the study period. Notably, the effectiveness of interventions remains evident even after accounting for the contribution of the cohort effect. Our projections of age-stratified prevalence up to 2023 suggest a downward trend across all age groups. However, those aged over 40 are expected to maintain a prevalence exceeding 1%, highlighting the imperative for ongoing sustained efforts toward disease elimination. The age-period-cohort modeling framework offers broad applicability in other liver fluke endemic regions, particularly where data on host reservoirs beyond humans is limited. This approach provides valuable insights for future public health planning and resource allocation, aiming to mitigate the disease burden in affected regions.”*

6. Line 72. Not just transmission to humans

>> We thank the reviewer for this comment. In response, we have replaced the phrase “*humans*” with “*carnivorous mammals, including humans*” in the revised manuscript.

*[P4 L72] “This food-borne trematode completes its life cycle through three reservoir hosts—freshwater snails, freshwater fish, and humans (or other carnivorous mammals)—with the consumption of raw or undercooked freshwater fish infected with C. sinensis metacercariae serving as the primary mode of transmission to carnivorous mammals, including humans.”*

7. Line 246 247. Is there evidence that age cohort has not been taken into consideration for interventions? It is my understanding that this is known and has been considered in current efforts.

>> We appreciate the reviewer’s insightful comments, which have greatly contributed to refining our manuscript. As the reviewer pointed out, targeted interventions that consider age (or cohort) aspects could potentially lead to an underestimation of intervention effects in our age-period-cohort modeling approach. This is because the cohort effect adjustment, which partially reflects the impact of such targeted interventions, could obscure their overall impact. However, in practice, disentangling the impact of such interventions on period and cohort effects is challenging as the implementation of interventions targeting specific age groups often coincides with broader interventions targeting the general population. For example, broad public health campaigns aiming at changing raw freshwater fish consumption have been implemented concurrently with screening interventions targeting those residing along the main river stream (typically older populations) in Korea [1–2]. Furthermore, despite the targeted interventions, the residual cohort effects cannot be negligible, and neglecting the incorporation of the cohort effects may still lead to biased results in either the evaluation of interventions or projections of future trajectories of the disease. We have now discussed these points in the revised manuscript.

*[P16 L339–L352] “Additionally, it is worth noting that our APC modeling approach may potentially underestimate the effectiveness of interventions specifically targeting high-risk cohort groups because adjusting for cohort effects can obscure the impact of these targeted measures. Indeed, a large-scale stool examination has been conducted in Korea since 2011 targeting residents along main river streams. Although this intervention was not directed at specific cohorts, its effects might be particularly pronounced among older generations due to their higher proportion in these areas. However, in practice, disentangling the specific impacts of such targeted interventions from period and cohort effects is challenging because these interventions often coincide with broader public health initiatives (e.g., mass health campaigns aiming at changing risk behaviors of C. sinensis infection). Moreover, even with targeted interventions, residual cohort effects due to the sustained raw freshwater fish consumption are highly likely to persist; thus, neglecting to account for the cohort effects may lead to biased estimates of intervention effectiveness and inaccurate projections of future disease trends.”*

[1] Yoo, Won Gi, Woon-Mok Sohn, and Byoung-Kuk Na. "Current status of Clonorchis sinensis and clonorchiasis in Korea: epidemiological perspectives integrating the data from human and intermediate hosts." Parasitology 149.10 (2022): 1296-1305.

[2] Oh, Jin‐Kyoung, et al. "Control of clonorchiasis in Korea: effectiveness of health education for community leaders and individuals in an endemic area." Tropical Medicine & International Health 19.9 (2014): 1096-1104.

8. Line 258 to 261. Should also comment on the first intermediate host as well.

>> We thank the reviewer for this comment. We now have mentioned the first intermediate host (snails) in the revised manuscript.

*[P13 L273–277] “For instance, despite comparable levels of raw freshwater fish consumption behavior to other regions, the reported prevalence in Gangwon province was notably low (****Fig S****2), attributable to the reported limited inhabitant area of the fish with C. sinensis infection in this region [29], as well as potentially differing ecological dynamics of the first intermediate host (snails).”*

9. Lines 269 to 273. Would modelling low and high risk regions (based on correlation mentioned) be more informative for providing recommendations for future intervention strategies? For example, would the model behave differently in high risk regions?

>> We thank the reviewer for raising this point. We strongly agree that applying this modeling approach to the high-endemic regions would provide more informative insights into controlling *C. sinensis* infection in Korea. Nevertheless, we have relied on national-level data in our study due to the limited duration of the high-endemic region survey data (2013–2021), which makes it challenging to accurately assess the cohort effect. We have now discussed the potential for future application of the age-period-cohort modeling approach to the high-endemic region data in the revised manuscript.

*[P17 L370–373] “Despite this study offering insights into the national-level situational assessment of C. sinensis infection, future studies applying the APC modeling approach to data from high-endemic regions with a longer observation period could provide more detailed guidance for developing future control strategies in Korea.”*
