# Supplementary material for: Age, period, and cohort effects of Clonorchis sinensis infection prevalence in the Republic of Korea: Insights and projections
Source: PLoS Negl Trop Dis. 2024 Oct 11;18(10):e0012574. doi: 10.1371/journal.pntd.0012574 (PMC11498711; doi:10.1371/journal.pntd.0012574)
Supplement: Attachment — Submitted filename: Response to Reviewers.docx [file pntd.0012574.s009.docx]

**Point-by-Point response**

**Reviewer 1**

1. After carefully reviewing the revised manuscript of the author, it is found that the author has carefully revised the comments and suggestions of the reviewer, only one issue should be address. 
L66-68, “Clonorchis sinensis (C. sinensis)...... including the Republic of Korea (hereafter “Korea”), mainland China, Taiwan, and northern Vietnam [1–3]” should be replaced with “Clonorchis sinensis (C. sinensis), a prevalent intestinal helminth primarily endemic to the East Asian countries, including the Republic of Korea (hereafter “Korea”), China, and Vietnam [1–3]”.
L298, Please delete Taiwan.
It is well known that Taiwan is a province of China, so it is not appropriate to put China and Taiwan together. It is suggested to delete the words "Taiwan" in these sentences.

>> In response to this comment, We have now removed “Taiwan” from the revised manuscript.

*[P4 L66–67] “Clonorchis sinensis (C. sinensis), a prevalent intestinal helminth primarily endemic to the East Asian region—including the Republic of Korea (hereafter “Korea”), China, and northern Vietnam—poses significant public health threats in this region.”*

*[P14 L315–P15 L318] “Nevertheless, this APC modeling approach holds broad applicability to other endemic regions to C. sinensis infection (e.g., China and northern Vietnam), serving as an effective tool for evaluating interventions and facilitating projections (or nowcasting) between survey iterations.”*

**Reviewer 3**

1. This study builded and applied the age-period-cohort modeling approach to nationally representative C. sinensis infection prevalence data from 1981–2012 in Korea, in order to provide valuable insights for future public health planning and resource allocation. This research has certain scientific value and reference significance, I agree to publish.

>> We thank the reviewer for the time and effort in providing valuable comments.
